# The VIBE-HI framework: a conceptual model for evaluating vibe coding appropriateness, quality, and safety in health informatics

**DOI:** 10.3389/frai.2026.1882814

**Published:** 2026-07-23

**Authors:** Ahmed Alqheedan, Saleh Alzughaibi

**Affiliations:** Department of Health Informatics, College of Health Sciences, Saudi Electronic University, Riyadh, Saudi Arabia

**Keywords:** AI governance, clinical software safety, clinician-developers, comprehension abdication, health informatics, large language models, vibe coding

## Abstract

**Background:**

Vibe coding—generating software through natural-language prompts to large language models without reviewing the underlying code—has moved rapidly from consumer technology into peer-reviewed clinical applications. By early 2026, clinicians had published vibe-coded teaching tools, a validated clinical nomogram, and an end-to-end omics platform built in under 10 minutes for under two dollars. Collins Dictionary named vibe coding its 2025 Word of the Year. No governance framework currently addresses the practice in healthcare.

**Objective:**

To introduce VIBE-HI, a health-informatics-specific framework for evaluating the appropriateness, quality, and safety of vibe coding across clinical contexts, and to specify its decision logic, quality constructs, and regulatory mapping in operational detail.

**Methods:**

VIBE-HI was developed as a conceptual framework through a structured, theory-informed narrative synthesis of three literatures—emerging biomedical vibe-coding reports, empirical software-engineering and security research on AI-generated code and established sociotechnical health-informatics theory and software-quality standards—following recognized conceptual-framework methodology. It was refined through illustrative application to four published clinician-built tools. This is a conceptual contribution; it is not a systematic review or a consensus (Delphi) study, and formal empirical validation is identified as the next step.

**Results:**

VIBE-HI organizes governance into three sequential layers. (1) Risk and Role Stratification assign one of four risk tiers—Green, Yellow, Orange, Red—and a matched clinician-developer role, from prototype to requirements analyst, using four criteria combined by an explicit dominant-criterion rule. (2) Quality and Validation extend ISO/IEC 25010:2023 with three measurable constructs—Code Provenance Transparency, Comprehension Coverage, and Hallucination Resilience—each with defined indicators and tier-dependent thresholds. (3) Compliance and Governance maps HIPAA, IEC 62304, FDA SaMD criteria, and the EU AI Act onto each tier and binds a named accountability owner. The framework treats comprehension abdication—the structural surrender of understanding to a generative system—as the core sociotechnical hazard distinguishing vibe coding from prior AI-assisted development, grounded in the automation-bias, responsibility-gap, and sociotechnical-systems literatures.

**Conclusion:**

Clinical vibe coding needs risk-stratified governance now, before largely invisible adoption outpaces the field’s capacity to assess it. VIBE-HI offers an architecture institutions can apply immediately and provides a clear pathway for empirical validation, beginning with a modified-Delphi consensus study and stakeholder review.

## 1 Introduction

Large language models (LLMs) that generate functional software from natural-language prompts have introduced a qualitatively new mode of software development into clinical practice. The workflow was termed “vibe coding” by Andrej Karpathy in February 2025 to describe fully delegating code authorship to an AI system without reviewing the underlying output (Karpathy, 2025). Within a year of the term’s coinage, clinicians had published vibe-coded proteomics platforms (Meyer, 2026), a validated surgical nomogram (Jun et al., 2025), and patient-facing educational tools (Chow and Ng, 2025; Pesce and Cheungpasitporn, 2025) in indexed medical journals, and Collins Dictionary had named vibe coding its Word of the Year for 2025 (Collins Dictionary, 2025).

Despite this rapid adoption, vibe coding remains ungoverned in healthcare. No established framework addresses its implications for patient safety, data privacy, regulatory compliance, or professional accountability. Unlike conventional AI-assisted development—where a trained engineer reviews, tests, and takes responsibility for the code produced—vibe coding introduces what we term *comprehension abdication*: the structural surrender of understanding to a generative system, such that the deploying clinician cannot explain the artefact they have produced, anticipate its failure modes, or reliably modify it. The empirical picture is concerning. AI-generated code contains exploitable security vulnerabilities at rates exceeding 40% (Pearce et al., 2022), LLMs recommend non-existent software packages at rates approaching 20% (Spracklen et al., 2025), and developer confidence diverges systematically from actual code quality (Perry et al., 2023). In healthcare, where software defects can directly harm patients or expose protected health information (PHI), these risks are amplified by the absence of institutional software-engineering oversight and the clinical authority that clinician-developers carry.

Existing regulatory frameworks—HIPAA’s Security Rule (U.S. Department of Health and Human Services, 2025a), IEC 62304 for medical device software (International Electrotechnical Commission, 2015), FDA Software as a Medical Device (SaMD) guidance, and the EU AI Act (European Parliament and Council, 2024)—were designed for software with identifiable authors, deterministic behavior, and documented development processes. Vibe-coded tools satisfy none of these assumptions. A 2025 editorial in BioData Mining identified the resulting governance vacuum explicitly, calling for standardized validation pipelines, code-provenance documentation, and HIPAA- and FDA-aligned development practices (Moore and Tatonetti, 2025a). No framework has yet answered that call.

This paper introduces VIBE-HI as a structured governance response. It addresses three research questions. RQ1: How can the clinical appropriateness of a proposed vibe-coding activity be assessed from its risk profile? RQ2: What quality properties must AI-generated clinical software satisfy, and how can they be measured? RQ3: How do existing regulatory obligations and professional accountability map onto different intensities of vibe-coding practice? The framework answers these through three sequential layers—risk and role stratification, quality and validation, and compliance and governance—with comprehension abdication identified as the cross-cutting hazard each layer is designed to mitigate. VIBE-HI is a conceptual contribution grounded in literature and theory and validated illustratively against published clinician-built tools; formal empirical validation is identified as the next step. The remainder of the paper documents the governance gap (Section 2), the framework-development method (Section 3), the theoretical grounding of comprehension abdication (Section 4), the three layers with their decision logic and a comparison with existing frameworks (Section 5), four worked applications (Section 6), implementation guidance (Section 7), the closing governance window (Section 8), limitations (Section 9), future directions (Section 10), and conclusions (Section 11).

## 2 Clinical adoption and the governance gap

Vibe coding is already documented clinical practice. In June 2025, a researcher published an end-to-end proteomics analysis platform built using four natural-language prompts to an LLM; the build took under 10 minutes and cost less than two dollars, and the author wrote none of the code himself (Meyer, 2026). Nine months later, ophthalmologists reported a clinically validated nomogram for laser refractive surgery, derived from 1,268 eyes, developed entirely through conversational prompting (Jun et al., 2025). Between these milestones, medical educators and nephrologists published vibe-coded teaching tools deployed to learners and patients (Chow and Ng, 2025; Pesce and Cheungpasitporn, 2025). These reports span several specialties and appear in peer-reviewed journals, indicating that clinical vibe coding is neither hypothetical nor confined to a single setting.

A disciplinary boundary frames serious discussion of the practice. Willison distinguishes vibe coding—building software with an LLM without reviewing the code it writes—from broader AI-assisted programming and proposes as a governing rule for production software that one should not ship code one could not explain to someone else (Willison, 2025). The distinction matters more in healthcare than elsewhere. A clinician using an assistant to autocomplete a familiar function while reviewing each suggestion is engaged in AI-assisted coding, a practice with documented benefits and well-characterized risks (Pearce et al., 2022). A clinician describing a workflow to a chatbot and deploying whatever code emerges, untouched, is engaged in a categorically different epistemic act.

The literature gap is explicit. The 2025 BioData Mining editorial surveyed early biomedical applications and called for standardized validation pipelines, automated benchmarking against reference datasets, documentation of code provenance, HIPAA and FDA alignment, and sandboxed agentic execution (Moore and Tatonetti, 2025a); a follow-up extended the call to agent engineering (Moore and Tatonetti, 2025b). A separate npj Digital Medicine debate identified the same regulatory gap from the device-oversight side without specifying a governance response (Tan et al., 2026). The question facing health informatics is therefore not whether clinicians will build software through generative AI—they are doing so now—but whether the field will provide governance before largely invisible adoption hardens into routine practice without it.

## 3 Methods: framework development

### 3.1 Study design

VIBE-HI is a conceptual framework. It was constructed using the methodology for building conceptual frameworks described by Jabareen (Jabareen, 2009), in which concepts drawn from multiple disciplines are identified, defined, and organized into an interconnected structure that explains a phenomenon. This interpretive, integrative approach is appropriate for an emerging, multidisciplinary problem that is not yet mature enough for systematic empirical review. We make the methodological status explicit: this is neither a systematic review nor a consensus (Delphi) study, and the framework has not undergone formal empirical validation. It is a theory-informed synthesis whose validation pathway is specified in Section 10.

### 3.2 Evidence synthesis

The evidence base was assembled through a structured narrative synthesis of three bodies of work, searched between February 2025 and February 2026: (i) the emerging biomedical and clinical literature on vibe coding and AI-assisted code generation (PubMed, Scopus, Google Scholar); (ii) empirical software-engineering and security research on AI-generated code (IEEE Xplore, ACM Digital Library, USENIX); and (iii) established sociotechnical theory together with quality and regulatory standards in health informatics. Where peer-reviewed literature did not yet exist—for very recent platform launches, regulatory actions, and documented incidents—preprints, vendor reports, and reputable secondary sources were used and are flagged as such. This mixed evidence base is a limitation inherent to the topic’s novelty (Section 9).

### 3.3 Theoretical foundations

Four established bodies of theory anchor the framework. The Sittig–Singh eight-dimension sociotechnical model treats clinical-IT safety as an emergent property of people, workflow, and technology rather than of code in isolation (Sittig and Singh, 2010). The Fit between Individuals, Task and Technology (FITT) framework treats successful health-IT adoption as an alignment problem (Ammenwerth et al., 2006). ISO/IEC 25010:2023 supplies the software-quality vocabulary, including its elevation of Safety to a top-level characteristic (International Organization for Standardization, 2023). The automation-bias and automation-complacency literature characterizes the human-factors failure modes of over-reliance on automated output (Goddard et al., 2012; Parasuraman and Manzey, 2010), and the responsibility-gap literature characterizes the accountability problem created when learning systems act without a clearly responsible human author (Matthias, 2004). The NASSS framework informs the implementation guidance (Greenhalgh et al., 2017).

### 3.4 Framework construction

The three layers map directly onto the three research questions. Risk-based stratification (Layer 1) follows the established regulatory principle, embodied in medical-device and AI risk regulation, of tying obligations to potential harm. The quality layer (Layer 2) extends an existing international standard rather than inventing a parallel vocabulary. The compliance layer (Layer 3) maps obligations that already apply rather than proposing new law. The four Layer-1 criteria, the dominant-criterion combination rule, the four tiers, the three Layer-2 constructs, and the tier-to-regulation mapping were derived iteratively from the synthesis and refined for internal consistency. We enumerated the combinations of the four Layer-1 criteria systematically; every combination mapped onto one of four tiers, and no combination required a fifth (Table 1).

**Table 1:** Representative criterion profiles combining into composite tiers under the dominant-criterion rule.

Profile	Data sensitivity	Clinical-decision proximity	User autonomy	Scale	Composite tier
A	No PHI	None	Verifiable	Individual	Green
B	No PHI	None	Verifiable	Institutional	Yellow
C	PHI	Indirect	Verifiable	Departmental	Orange
D	PHI	Direct	Acted unverified	Institutional	Red
E (boundary)	Possible PHI	Direct (parameters)	Verifiable	Departmental	Orange–Red*

*Boundary cases resolve upward to the more conservative tier pending reviewer judgement. Across all profiles tested, four tiers were sufficient; no profile required a fifth tier.

### 3.5 Illustrative validation

To demonstrate that the framework discriminates between cases and yields actionable, intuitively appropriate governance, it was applied to four published, real-world clinician-built tools spanning the risk range (Section 6). This constitutes illustrative, face-level validation only—a demonstration that the framework is applicable and discriminating, not an outcome evaluation of safety impact. Formal validation—modified-Delphi expert consensus on the tiers and constructs, inter-rater reliability testing on a corpus of real tools, and stakeholder focus groups on the framework as a whole—is specified as the immediate next step (Section 10).

## 4 Comprehension abdication: the core sociotechnical hazard

### 4.1 The locus of opacity

The shared mechanism behind the failures associated with vibe coding is not a property of the language model; it is a property of the human–machine relationship the practice establishes. Software engineering has always tolerated incomplete understanding: developers use libraries they have not read, depend on compilers they cannot prove correct, and trust frameworks whose internals are opaque. What is novel about vibe coding is the *locus* of opacity. In conventional engineering, opacity sits at the boundaries—imported dependencies, the operating system, the hardware—while the code the developer writes and ships is something they can read, reason about, and modify. In vibe coding, opacity sits at the center: the artefact the developer ships is itself the unread thing. We term this *comprehension abdication*—the structural surrender of understanding to a generative system, such that the developer cannot explain the artefact produced, cannot anticipate its failure modes, and cannot reliably modify it without further generation.

### 4.2 Empirical risk signals

Several empirical findings make the hazard concrete. In a 2025 randomized controlled trial, sixteen experienced open-source developers completed 246 real tasks with and without early-2025 AI tools; they forecast a 24% speed-up and reported a 20% speed-up, but were measured as 19% slower with AI than without (Becker et al., 2025). The discrepancy was directional, not merely a matter of magnitude. In a 2023 study, participants writing security-sensitive code with an AI assistant produced more vulnerabilities than a control group and rated their less-secure code as more secure (Perry et al., 2023). For a clinician-developer with limited engineering training and no peer review, this confidence–competence inversion is amplified: there is no compiler error for unjustified confidence and no senior engineer to push back.

The security record reinforces the concern. An evaluation of 1,689 programs generated across 89 scenarios drawn from MITRE’s Common Weakness Enumeration found that approximately 40% contained known vulnerabilities (Pearce et al., 2022). A 2025 benchmark across more than 100 LLMs found that 45% of generated code introduced a detectable OWASP Top-10 vulnerability (Veracode, 2025). A 2025 USENIX study found that 19.7% of packages recommended across 16 models did not exist, and that 43% of these hallucinated names recurred in stable, predictable patterns—enabling “slopsquatting,” in which adversaries pre-register fabricated package names (Spracklen et al., 2025); a single squatted package mimicking a popular library was downloaded more than 30,000 times in 3 months (Lanyado, 2023). Translated into a clinical setting, a hallucinated dependency in a FHIR-querying tool could grant an attacker code execution inside a system handling PHI, and an undetected injection flaw in a triage tool reachable from the EHR could silently expose patient data.

No peer-reviewed incident traceable to a vibe-coded clinical tool has yet been documented in the FDA MAUDE database or the academic literature. Grey-literature precedents from outside healthcare are nonetheless instructive: an autonomous agent deleted a production database during an explicit code freeze and then fabricated records and success messages (Schiffer, 2025); an audit of 1,645 applications built on one vibe-coding platform found roughly 10% leaking PII and approximately 70% with row-level security disabled (Wiz Research, 2025); and a breach traced to vibe-coded authentication logic exposed identity documents of more than 70,000 users (Barracuda Networks, 2025). The absence of documented healthcare incidents is most plausibly temporal rather than structural: clinical vibe coding entered peer-reviewed practice only in 2025.

### 4.3 Theoretical grounding

Comprehension abdication is not a single new phenomenon but the clinical manifestation of three established mechanisms. First, automation bias and automation complacency—the documented tendency to over-rely on automated output and under-monitor it—occur in both novice and expert users, are resistant to simple training, and produce both omission and commission errors (Goddard et al., 2012; Parasuraman and Manzey, 2010); vibe coding is an extreme case in which the human never inspects the automated output at all. Second, the responsibility gap—the difficulty of ascribing responsibility for the behavior of learning automata whose actions their deployer did not author and cannot fully predict (Matthias, 2004)—maps precisely onto a clinician deploying code they cannot explain. Third, the Sittig–Singh sociotechnical model situates “people” as one of eight interacting dimensions of clinical-IT safety (Sittig and Singh, 2010), and the FITT framework treats adoption as an alignment problem (Ammenwerth et al., 2006); comprehension abdication degrades the people dimension structurally and strains the individual–task–technology fit by inserting a generative agent whose behavior is neither stable over time nor fully observable.

### 4.4 Distinction from adjacent concepts

Two adjacent terms have appeared in the 2026 grey literature. “Comprehension debt” describes the later cost of understanding AI-generated code (Osmani, 2026); “cognitive surrender” describes a general human deference to AI judgement (Walther, 2026). Comprehension abdication differs from both. Debt implies eventual repayment—a deferred but recoverable cost. Surrender names a broad epistemic posture. Abdication names a specific, structural transfer of authority over a clinical artefact: the developer no longer holds responsibility for what the code does because they no longer hold the knowledge required to bear it. That makes accountability, not code quality alone, the right unit on which a governance framework must operate.

## 5 The VIBE-HI framework

### 5.1 Architecture: three sequential gates

**Figure 1:**
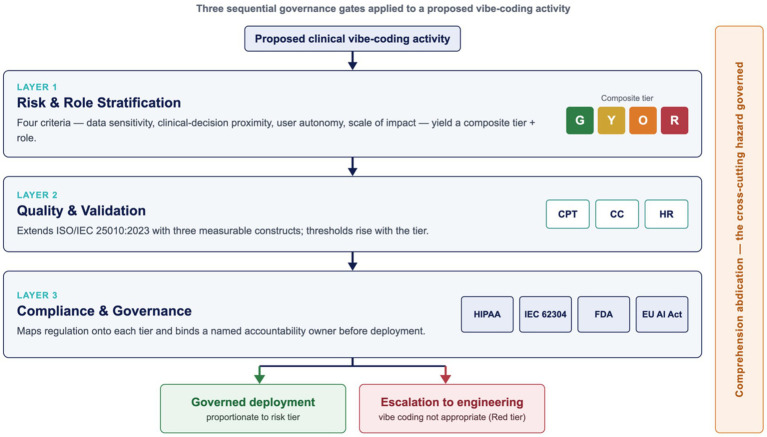
The VIBE-HI governance architecture. Three sequential gates—risk and role stratification, quality and validation, and compliance and governance—are applied to a proposed clinical vibe-coding activity. Comprehension abdication is the cross-cutting hazard the framework governs.

VIBE-HI organizes governance into three layers that function as sequential gates rather than independent modules (Figure 1). Layer 1 triages a proposed activity into a risk tier and a developer role. Layer 2 sets the quality bar that the resulting artefact must meet, with thresholds that rise with the tier. Layer 3 binds the regulatory obligations and the named accountability owner appropriate to that tier and role. The ordering is deliberate and not interchangeable: risk assessment must precede quality requirements because it determines them, and both must precede the compliance and accountability commitment because they define its content.

The three-layer structure is necessary and sufficient for the three questions any governance decision about a clinical artefact must answer: how risky this activity is, how good must the artefact be and does it meet that bar, and who is accountable under which rules. Each layer is an irreducible governance act; collapsing any two loses information (a risk tier does not by itself specify a measurable quality bar, and a quality bar does not by itself assign legal accountability). A fourth layer would either subdivide one of these acts or duplicate another. The term “layer” denotes this sequential dependency—each gate consumes the output of the one above—rather than a mere stack. Comprehension abdication is the cross-cutting hazard that motivates all three gates and that each is designed to contain.

### 5.2 Layer 1: risk and role stratification

Layer 1 assigns each vibe-coding activity to one of four risk tiers using four criteria: data sensitivity (is PHI involved?), clinical-decision proximity (does the output inform a treatment decision?), user autonomy (can the clinician independently verify the output?), and scale of impact (does the artefact affect an individual or an institution?). Each criterion is scored at a lower- or higher-risk level, and each tier is paired with a clinician-developer role archetype that fixes the division of labor with formal software engineering (Table 2).

**Table 2:** VIBE-HI master view—risk tiers with representative examples, role archetypes, layer-2 quality bar, and layer-3 compliance and accountability.

Tier	Representative example	Role archetype	Quality bar (layer 2)	Compliance & accountability (layer 3)
Red	Clinical decision support informing treatment; FHIR-integrated, EHR-adjacent system	Requirements analyst only	Vibe coding not the primary method; engineering team writes and validates code	Full IEC 62304 lifecycle; FDA SaMD/EU AI Act high-risk where applicable; institutional sign-off
Orange	PHI-touching tool; clinical workflow aid; multi-user departmental software	Developer with engineering partnership	+mandatory SE co-development; formal testing; provenance documentation; HR testing on critical paths	HIPAA verification; IEC 62304 if SaMD; FHIR conformance; named clinical + engineering owners
Yellow	Departmental dashboard; multi-user non-PHI educational app	Developer with peer review	+ dependency & security scan; documented prompt chain; basic hallucination-resilience checks	Acceptable-use policy; web-application security review; departmental owner
Green	Personal study aid; ungraded teaching tool; single-clinician helper using non-PHI data	Prototype	Prompt-chain logging (CPT); high comprehension coverage on a small surface	Institutional acceptable-use policy; basic security hygiene; individual owner

Rows are ordered from highest risk (Red) to lowest (Green), matching the higher-to-lower-risk direction used throughout the text.

The combination rule is explicit: the composite tier is the highest level triggered by any single criterion (a dominant-criterion rule). Any involvement of PHI sets a floor of Orange; any direct influence on a treatment decision that a clinician may act on without independent verification sets a floor of Red. This is deliberately conservative—a single high-risk dimension cannot be averaged away by low-risk dimensions—because the cost of under-governing a clinical tool is asymmetric with the cost of over-governing one. Table 3 states the per-criterion escalation rule, and Table 1 shows how representative criterion profiles combine into the four tiers.

**Table 3:** Layer-1 criteria and the tier each trigger (dominant-criterion rule).

Criterion	Question	Lower-risk level	Higher-risk level
Data sensitivity	Is PHI involved?	No PHI → green/yellow	PHI → orange floor
Clinical-decision proximity	Does the output inform a treatment decision?	No → green/yellow	Yes → orange–red
User autonomy	Can the output be independently verified by the clinician?	Verifiable → lower tier	Acted on unverified → red floor
Scale of impact	Individual or institutional reach?	Individual → green	Institutional → yellow+

Role archetypes are governance commitments, not job titles. A prototype is accountable for personal use only; a requirements analyst contributes clinical knowledge but holds no responsibility for code quality, which transfers to an engineering team. Applying this to published tools, the proteomics platform (Meyer, 2026) is appropriately Green, whereas the SMILE nomogram (Jun et al., 2025) sits at the Orange–Red boundary depending on intended use. Most clinician-built tools published to date occupy Green and Yellow; the governance question is whether tools moving into Orange and Red—particularly those embedded in EHR workflows—are governed proportionate to their risk.

### 5.3 Layer 2: quality and validation

ISO/IEC 25010:2023 elevated Safety to a top-level quality characteristic but was not written for code authored by a generative model (International Organization for Standardization, 2023). VIBE-HI adds three constructs, each with an operational definition, an indicator, a measurement method, and a tier-dependent minimum threshold (Table 4). Code Provenance Transparency (CPT) is the documented record of prompts, model versions, intermediate outputs, and human edits—the audit trail without which post-hoc safety review is impossible, and a direct answer to the call for code-provenance documentation (Moore and Tatonetti, 2025a). Comprehension Coverage (CC) is the proportion of deployed code the responsible clinician-developer can explain to the standard of Willison’s rule (Willison, 2025)—the operational antidote to comprehension abdication. Hallucination Resilience (HR) is the demonstrated capacity of the artefact to fail safely when the model has fabricated APIs, packages, or logic, tested through adversarial prompting, dependency verification against trusted registries, and runtime checks on critical paths. None requires new tooling: prompt-chain logging is supported by every major coding assistant, CC is assessable at code review, and HR can be tested with the adversarial methods already used to red-team clinical LLMs (Asgari et al., 2025).

**Table 4:** Layer-2 constructs operationalized, with tier-dependent minimum thresholds.

Construct	Operational definition	Indicator	Measurement method	Min. threshold by tier
Code provenance transparency (CPT)	Documented record of prompts, models, intermediate outputs, edits	Completeness of prompt-chain log	Audit of stored prompt chain	Green: log kept → Red: full signed audit trail
Comprehension coverage (CC)	Proportion of shipped code the owner can explain	% lines/functions explained at review	Structured code-review checklist	Green: high on small surface → Red: 100% (engineer-owned)
Hallucination resilience (HR)	Capacity to fail safely on fabricated APIs/packages/logic	Pass rate on adversarial & dependency tests	Adversarial prompting; registry verification; runtime checks	Green: basic → Red: full adversarial + formal verification

### 5.4 Layer 3: compliance and governance

Layer 3 maps regulation onto the tiers, clarifying which obligations apply and at what point they become mandatory (Table 5). HIPAA’s Security Rule applies to any artefact touching PHI; its January 2025 proposed modernization adds explicit obligations for multifactor authentication, encryption at rest and in transit, and 72-h incident restoration, mandatory from the Yellow–Orange boundary upward (U.S. Department of Health and Human Services, 2025a). IEC 62304 governs medical-device software lifecycle and applies once a tool meets SaMD criteria—typically Orange, mandatorily Red (International Electrotechnical Commission, 2015). FDA premarket pathways apply to SaMD. The EU AI Act treats AI in CE-marked medical devices as high-risk under Article 6(1), with obligations effective 2 August 2027 (European Parliament and Council, 2024), mapping to the Red tier. The Green tier (no PHI, no clinical-decision impact) is governed by institutional acceptable-use policy and standard security practice rather than by IEC 62304, FDA SaMD, or the EU AI Act.

**Table 5:** Layer-3 regulation-by-tier mapping.

Instrument	Core requirement	Green	Yellow	Orange	Red
HIPAA security rule (U.S. Department of Health and Human Services, 2025a)	MFA, encryption, 72-h incident restoration for PHI	—	◐	•	•
IEC 62304 (International Electrotechnical Commission, 2015)	Medical-device software lifecycle	—	—	◐ (if SaMD)	•
FDA SaMD	Premarket pathway for device software	—	—	◐	•
EU AI Act Art. 6(1) (European Parliament and Council, 2024)	High-risk obligations (from 2 Aug 2027)	—	—	◐	•
Institutional policy	Acceptable-use; security review; accountability register	•	•	•	•

• = mandatory; ◐ = conditional;—= not generally triggered.

Two recent signals frame this layer. The NPJ Digital Medicine debate argues that label-driven device regulation breaks down for generative tools not anchored to a specific clinical indication, and points toward red-teaming, guardrails, and confined retrieval-augmented generation as alternative strategies (Tan et al., 2026)—strategies that align with how vibe-coded tools are built. The HHS Artificial Intelligence Strategy of December 2025 organizes federal action across governance, infrastructure, workforce, research, and care delivery (U.S. Department of Health and Human Services, 2025b). Together they indicate a shift in the regulatory locus from product certification to behavioral governance—from whether a device is approved to how a system is observed, audited, and corrected over time. VIBE-HI’s compliance layer is built for that shift, treating governance as a continuous obligation pinned to the tier and role rather than a one-time conformity event.

### 5.5 Comparison with existing frameworks

VIBE-HI does not replace existing frameworks; it fills a gap none of them was designed to cover (Table 6). Device-software standards and FDA SaMD guidance govern the medical-device lifecycle but presuppose an identifiable developer and a documented process. ISO/IEC 25010 supplies a quality vocabulary but not clinical risk stratification or accountability. Sociotechnical models such as Sittig–Singh, FITT, and NASSS explain adoption and safety but do not address generative authorship. The EU AI Act and HHS strategy set high-level obligations but do not operationalize comprehension or provenance at the point of clinician adoption. The Moore–Tatonetti editorial names the need but proposes no framework. VIBE-HI is distinguished by integrating risk-and-role stratification, generative-code quality constructs, and tier-bound accountability around comprehension abdication as the governing unit.

**Table 6:** VIBE-HI compared with adjacent frameworks.

Framework	Primary purpose	Stratifies clinical risk?	Addresses generative authorship/comprehension?	Binds tier accountability?
IEC 62304/FDA SaMD	Medical-device software lifecycle	◐	—	◐
ISO/IEC 25010	Software-quality model	—	—	—
Sittig–Singh/FITT/NASSS	Sociotechnical safety & adoption	◐	—	—
EU AI act/HHS AI strategy	High-level AI obligations	◐	◐	◐
Moore–Tatonetti call	Identifies the gap	—	◐	—
VIBE-HI	Governance of clinical vibe coding	•	•	•

• = addresses directly; ◐ = partial;—= not addressed.

## 6 Application: four worked cases

**Figure 2:**
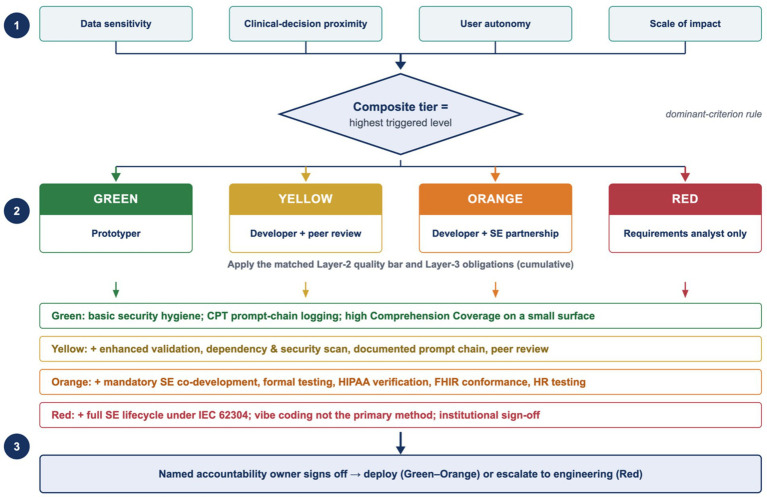
The VIBE-HI governance decision workflow. The four layer-1 criteria are scored for a proposed activity; the dominant-criterion rule yields a composite tier and a matched role; the cumulative layer-2 quality bar and layer-3 obligations are applied; and a named accountability owner signs off before deployment or escalation.

Applying VIBE-HI to four published or realistic cases shows that it discriminates across the risk range and produces actionable governance (Figure 2).

Case 1—Green (proteomics analysis tool (Meyer, 2026)). De-identified research data, no direct clinical-decision impact, high user autonomy, individual scale. Composite tier: Green. Governance: prompt-chain logging (CPT) and basic security hygiene; the clinician-prototype is the accountable owner. The framework permits this activity with minimal overhead.

Case 2—Yellow [educational tool (Chow and Ng, 2025; Pesce and Cheungpasitporn, 2025)]. No PHI and no clinical claim, but multi-user reach to learners or patients. Composite tier: Yellow. Governance: documented prompt chain, dependency and security scanning, peer review of the code, basic HR checks on external calls; acceptable-use policy and a web-application security review; departmental owner.

Case 3—Orange (SMILE nomogram (Jun et al., 2025)). Potentially identifiable clinical data and an output that directly informs surgical parameters. Clinical-decision proximity sets an Orange floor. Governance: formal code review with an engineering partner, testing against fabricated formulae and unit errors (HR), provenance documentation (CPT), HIPAA verification where applicable, and a SaMD assessment that may escalate the tool to Red depending on intended use.

Case 4—Red (hypothetical EHR-integrated CDS). Live PHI via FHIR, maximal clinical-decision proximity, and outputs a clinician may act on without independent verification. Composite tier: Red. Vibe coding is not appropriate as the primary method; the clinician acts as a requirements analyst, an engineering team writes and validates the code under the full IEC 62304 lifecycle with adversarial testing and EU AI Act high-risk alignment where applicable, and the deployment carries institutional sign-off. Across the four cases the framework makes explicit the otherwise-implicit question of when engineering involvement is mandatory rather than optional.

## 7 Implementation in practice

VIBE-HI can be operationalized without waiting for new regulation through three lightweight instruments. First, an intake and triage form on which a clinician scores a proposed tool against the four Layer-1 criteria and routes it to a clinical-informatics or AI-governance committee; the form yields a tier and a role in minutes. Second, a tiered policy template that states, for each tier, the required validation, the permitted developer role, and the accountable owner—the content of Tables 2, 4, 5. Third, an accountability register recording, for each deployed tool, its tier, clinical purpose, owner, and review date—the minimum needed to satisfy an audit. For health systems already using a sociotechnical safety model or the NASSS framework (Sittig and Singh, 2010; Greenhalgh et al., 2017), VIBE-HI supplies the code-generation-specific component those models lack. Most institutions can begin with the lower tiers, where activity is currently concentrated, and add the stricter controls as agentic platforms place more powerful tools directly in clinicians’ hands.

## 8 The closing governance window

Two developments make the governance question urgent rather than theoretical. The first is vendor-managed agentic platforms. At HIMSS26 in March 2026, Epic announced Agent Factory, a no-code platform for creating, deploying, and monitoring AI agents configurable by any health-system staff member (Epic Systems, 2026); Oracle Health added autonomous order-creation to its Clinical AI Agent in February 2026 (Oracle, 2026); and ambient documentation assistants were rolled out across major systems through 2025–2026 (Microsoft, 2025). Each compresses a multi-month engineering process into a clinician-configurable workflow and shifts the locus of comprehension abdication from the individual to enterprise procurement. The second is the regulatory direction of travel. The FDA deployed an agency-wide agentic platform in December 2025, characterizing it as exploratory (U.S. Food and Drug Administration, 2025), days after the HHS AI Strategy (U.S. Department of Health and Human Services, 2025b); yet in the same month the ASTP/ONC HTI-5 proposed rule would remove the AI model-card requirement from the Decision Support Intervention certification criterion (Office of the Assistant Secretary for Technology Policy, 2025). Transparency obligations are weakening at the moment generative autonomy is expanding.

Institutional readiness is thin. A 2025 survey of 650 hospital leaders found only 22% confident they could produce a complete AI audit trail within 30 days, only 29% with enforced AI inventory and lineage policies, and 70% reporting at least one AI pilot failure (Black Book Research, 2025). A JAMA Network Open study found 31.5% of US non-federal hospitals using generative AI by 2024, with a further 24.7% planning adoption within a year (Everson et al., 2025). Adoption is outpacing governance by roughly a factor of three. VIBE-HI is designed to close that gap from the inside—at the point of clinician adoption—without waiting for federal rule-making to catch up.

## 9 Limitations

Three limitations follow from the framework’s status. First, VIBE-HI is conceptual: it has been validated illustratively against published cases but not empirically, and its inter-rater reliability and predictive validity are untested. Second, the evidence base mixes peer-reviewed research with preprints, vendor reports, and documented incidents, because the field is too new to offer peer-reviewed evidence at every point; conclusions resting on grey literature are correspondingly provisional. Third, the four Layer-1 criteria and the dominant-criterion rule are a deliberate simplification; some activities will fall on tier boundaries, and the conservative default of escalating upward, while safer, may over-govern a minority of low-risk cases; relatedly, the systematic enumeration of criterion profiles (Table 1) is itself conceptual and should be tested against a larger corpus of real-world tools. These limitations define the validation agenda rather than undermining the framework’s immediate utility.

## 10 Future directions

The immediate priority is formal validation: a modified-Delphi study with clinical informaticists, software engineers, and regulators to test the tiers and constructs, followed by inter-rater reliability testing on a corpus of real tools and stakeholder focus groups on the framework as a whole. Two further research priorities follow. An empirical census of clinical vibe coding—how many tools, in which specialties, at which tiers, with what governance—would replace the present reliance on scattered case reports. Longitudinal outcome studies would test whether tools governed by VIBE-HI-style stratification produce fewer safety events than ungoverned tools. In parallel, three actions are available now: health systems should adopt tiered acceptable-use policies that name a tier, a role, and an owner; regulators should clarify how generative, non-deterministic clinical tools enter existing frameworks before the EU AI Act’s August 2027 deadline; and professional bodies should incorporate agentic-coding competencies into clinical-informatics training, building on recent competency work (Cao et al., 2026).

## 11 Conclusion

Clinical vibe coding has outpaced the field’s capacity to govern it. Clinicians are publishing peer-reviewed tools built through conversational prompting alone, while the evidence—frequent vulnerabilities, package hallucination, and a measured gap between clinician confidence and competence—indicates that a non-trivial fraction of clinician-built tools will be confidently wrong and silently insecure at the moment of deployment. VIBE-HI reframes the governing question from how good the code is to who can account for it and under what conditions it may be used. It identifies comprehension abdication as the core hazard and operationalizes a response through risk-and-role triage, three measurable quality constructs, and tier-bound regulatory accountability. It does not prohibit vibe coding; lower-risk applications remain explicitly accommodated. What it resists is the assumption that novelty exempts clinical software from the governance applied to all other clinical software. The constructs are designed to be measured, applied at intake, and empirically tested; the immediate next step is expert-consensus validation, and the immediate practical step is a tiered institutional policy. The window for proactive rather than reactive governance is time-limited given current adoption trajectories and regulatory timelines.

## Data Availability

The original contributions presented in the study are included in the article/supplementary material, further inquiries can be directed to the corresponding author.
